# Construction of a predictive model for the efficacy of enhanced external counterpulsation therapy in patients with heart failure with preserved ejection fraction based on automated machine learning and echocardiography

**DOI:** 10.3389/fcvm.2026.1876579

**Published:** 2026-09-09

**Authors:** Xuan Zhang, Fuqiang Zeng, Jing Liu, Min Zeng, Li Zhou, Xin Wang

**Affiliations:** 1 Department of Ultrasound Medicine, ChongQing University FULing Hospital, Chongqing, China; 2 Department of Gynaecology and Obstetrics, ChongQing University QianJiang Hospital, Chongqing, China

**Keywords:** automated machine learning, echocardiography, efficacy prediction, enhanced external counterpulsation, heart failure with preserved ejection fraction, SHAP interpretability analysis

## Abstract

**Objective:**

This study aimed to integrate baseline clinical characteristics and multi-dimensional echocardiographic parameters of patients with heart failure with preserved ejection fraction (HFpEF), construct and validate an individualized predictive model for the efficacy of enhanced external counterpulsation (EECP) therapy in HFpEF patients based on an automated machine learning (AutoML) framework, and develop a corresponding clinical decision support system.

**Methods:**

A retrospective cohort study design was adopted, consecutively enrolling 550 HFpEF patients who received complete EECP treatment at the cardiovascular center of two tertiary grade-A hospital between January 2018 and December 2023. An AutoML predictive framework driven by an improved Newton's downhill optimizer (INDO) was constructed, automatically performing joint optimization of feature subset selection, model algorithm selection, and hyperparameter configuration under a rigorous double-layered nested cross-validation strategy. Five benchmark models, including logistic regression, support vector machine, adaptive boosting, extreme gradient boosting, and light gradient boosting machine, were simultaneously established for performance comparison. Multi-dimensional evaluation was conducted using the area under the receiver operating characteristic curve (ROC-AUC), area under the precision-recall curve (PR-AUC), accuracy, sensitivity, specificity, F1 score, Brier score, and decision curve analysis. The robustness of features was verified through LASSO regression, and the SHAP framework was employed for model interpretability analysis.

**Results:**

Among the 550 HFpEF patients, significant differences were observed between the treatment-effective group and the treatment-ineffective group in terms of age, body mass index, left ventricular ejection fraction, left atrial volume index, and E/e' ratio. The AutoML model optimized based on INDO achieved a ROC-AUC of 0.9254, a PR-AUC of 0.8425, an F1 score of 0.8285, and a Brier score of 0.1206 on the independent test set, with all metrics outperforming the five benchmark models. Decision curve analysis demonstrated that the model yielded a positive net clinical benefit within a threshold probability range of 16% to 96%. SHAP interpretability analysis revealed the 6-minute walk distance (6MWD), left ventricular ejection fraction (LVEF), age (Age), left atrial volume index (LAVI), E/e' ratio, and body mass index (BMI) as key predictors influencing the efficacy of EECP therapy.

**Conclusion:**

The predictive model constructed by integrating echocardiographic parameters and clinical features based on the AutoML framework driven by the INDO optimization algorithm can relatively accurately predict the individualized efficacy of EECP therapy in HFpEF patients, providing a interpretable, and clinically actionable intelligent assessment tool for indication screening and individualized decision-making regarding EECP therapy in HFpEF patients.

## 1 Introduction

Heart failure with preserved ejection fraction (HFpEF) is a clinical syndrome characterized by impaired left ventricular diastolic function as the core pathophysiological feature, currently accounting for more than half of all heart failure cases (1). With the acceleration of global population aging and the continuous rise in the burden of comorbidities such as hypertension, obesity, and diabetes, the incidence of HFpEF has been increasing year by year, making it a major public health issue that seriously threatens human health (2). Unlike heart failure with reduced ejection fraction (HFrEF), HFpEF exhibits more prominent clinical heterogeneity, with its pathogenesis involving multiple intertwined molecular pathways including systemic inflammatory response, microvascular endothelial dysfunction, cardiomyocyte stiffness, and interstitial fibrosis, leading to a high degree of variability among different patients in terms of clinical manifestations, disease outcomes, and treatment responses (3, 4). At the level of therapeutic strategies, in addition to conventional pharmacotherapy, enhanced external counterpulsation (EECP), as a non-invasive adjunctive circulatory support modality, has gradually attracted attention in recent years for its application value in HFpEF patients (5, 6). By sequentially compressing the lower limbs and buttocks during cardiac diastole, EECP increases venous return and coronary perfusion pressure while reducing cardiac afterload, thereby improving subendocardial myocardial perfusion and promoting collateral circulation establishment. Clinical studies have demonstrated that standardized EECP treatment can effectively improve exercise tolerance and cardiac functional class in HFpEF patients, with a subset of patients achieving significant clinical benefits (7–9). However, EECP treatment is characterized by a relatively long treatment cycle and high requirements for patient compliance, and not all HFpEF patients can achieve ideal therapeutic outcomes, with significant inter-individual differences in treatment efficacy. Therefore, accurately identifying the patient subgroups most likely to benefit from EECP prior to treatment is of great clinical significance for optimizing healthcare resource allocation and formulating individualized treatment plans, and constructing a precise efficacy prediction model represents the core technical pathway to achieve this goal.

Currently, research on predicting the efficacy of EECP treatment in HFpEF patients remains at an early stage. The predictive factor analyses proposed by previous scholars have mostly been limited to univariate regression or traditional multivariate logistic regression, and the candidate variables included have largely focused on demographic characteristics and a few clinical indicators, lacking systematic integration and quantitative assessment of echocardiographic parameters (10, 11). In fact, echocardiography, as the non-invasive imaging gold standard for evaluating cardiac diastolic function, provides parameters such as left atrial volume index, E/e' ratio, and left ventricular ejection fraction, which can reflect left ventricular filling pressure, the degree of diastolic dysfunction, and atrial remodeling status from different dimensions. These indicators are precisely closely related to the hemodynamic mechanism of action of EECP and thus possess potential value for predicting treatment efficacy. Furthermore, existing prediction studies have typically constructed prediction rules based on linear assumptions, making it difficult to capture the complex nonlinear dependencies and higher-order interaction effects that exist between baseline characteristics, cardiac structural and functional parameters, and EECP treatment response in HFpEF patients, which to a certain extent limits further improvement in prediction accuracy.

In recent years, the rapid development of machine learning techniques has opened up new technical pathways for overcoming the limitations of traditional parametric models (12). Compared with conventional methods such as logistic regression, machine learning algorithms do not rely on preset variable distribution assumptions and linear function constraints. They are capable of adaptively learning the intrinsic complex structure of data from high-dimensional clinical feature spaces and effectively identifying nonlinear relationships and interaction effects among variables. This characteristic is highly congruent with the practical needs of predicting treatment efficacy in a heterogeneous disease such as HFpEF. In particular, the proposal of the automated machine learning (AutoML) framework, which adaptively accomplishes joint optimization of feature subset selection, model selection, and hyperparameter configuration through algorithm-driven search strategies, overcomes the limitations of traditional machine learning research that overly relies on manual feature engineering and preset model architectures, enabling the approximation of globally optimal model configuration solutions under limited computational resources (13, 14). The value of multivariable, data-driven frameworks in enabling predictive, preventive, and personalized medicine has been increasingly recognized, particularly for long-term risk assessment in patients with comorbid conditions (15, 16). However, in the current field of EECP efficacy prediction for HFpEF patients, there remains a lack of sufficient exploration regarding the organic integration of the AutoML framework with rigorous cross-validation strategies and interpretability analysis tools to systematically construct a predictive model that combines high accuracy with clinical interpretability.

Based on the above research background, this study aims to take a cohort of HFpEF patients receiving EECP treatment as the research object, integrate baseline clinical characteristics and multi-dimensional echocardiographic parameters of patients, and construct an individualized predictive model for EECP treatment efficacy based on an optimized AutoML framework. It is expected to provide a interpretable, and clinically actionable intelligent assessment tool for indication screening of EECP treatment in HFpEF patients, thereby contributing to the optimization of individualized treatment decisions and long-term management strategies for this special population.

## 2 Methods

### 2.1 Study population

This study adopted a retrospective cohort study design, collecting data on HFpEF patients who received EECP treatment at the cardiovascular center of two tertiary grade-A hospital between January 2018 and December 2023. A total of 550 study subjects were obtained through inclusion and exclusion criteria. All study data were derived from the hospital electronic medical record system. As this was a retrospective study, patient informed consent was waived, and the study was reviewed and approved by the hospital ethics committee (ethics approval number: 2025-97). Inclusion criteria: ① Age 50–85 years; ② Meeting the clinical diagnostic criteria for HFpEF (left ventricular ejection fraction ≥50%, with evidence of diastolic dysfunction) (17); ③ Completion of a full course of EECP treatment; ④ Completion of comprehensive assessment before and after treatment; ⑤ Complete follow-up data. Exclusion criteria: ① Presence of contraindications to EECP treatment (aortic regurgitation, aortic dissection, active thrombophlebitis, etc.); ② Concomitant severe valvular heart disease requiring surgical intervention; ③ Left ventricular ejection fraction <50%; ④ Acute decompensated heart failure; ⑤ Loss to follow-up or incomplete clinical data.

### 2.2 Data collection and processing

Through the hospital electronic medical record system and picture archiving and communication system, data were collected including patient demographic characteristics (age, sex, body mass index), clinical characteristics (duration of heart failure, New York Heart Association functional class, 6-minute walk distance), laboratory tests (N-terminal pro-B-type natriuretic peptide), echocardiographic parameters (left ventricular ejection fraction, left ventricular mass index, left atrial volume index, E/e' ratio, tricuspid regurgitation velocity), treatment characteristics (number and duration of EECP treatment sessions), and concomitant medication use (angiotensin-converting enzyme inhibitors/angiotensin II receptor blockers, beta-blockers, mineralocorticoid receptor antagonists, sodium-glucose cotransporter 2 inhibitors). The primary outcome variable was EECP treatment effectiveness, defined as post-treatment improvement in cardiac function by ≥1 NYHA class or an increase in 6-minute walk distance of ≥30 meters (18). These two criteria were evaluated independently; when the two criteria yielded discordant results (one met while the other did not), the outcome was still classified as effective, consistent with the pre-specified “either-or” composite definition. NYHA class assessment was performed by two board-certified cardiologists who were blinded to the treatment allocation and the patient's 6MWD results, based on standardized clinical interview and physical examination. Any disagreement between the two assessors was resolved through discussion with a third senior cardiologist. Baseline 6MWD was confirmed to be comparable between the training and test sets, and the ≥30 m absolute improvement threshold was selected based on the established minimal clinically important difference (MCID) for patients with heart failure, which has been validated across a range of baseline functional capacities (18).

Missing value processing: This study adopted a multi-strategy approach to handle missing data. For variables with a missing proportion ≤3%, mean imputation (continuous variables) or mode imputation (categorical variables) was applied; for variables with a missing proportion of 3%–5%, multiple imputation was used (creating 5 imputed datasets). All missing value processing was performed independently in the training and test sets to avoid information leakage. The overall missing rate was approximately 2.10%, consistent with the actual data quality of clinical research.

### 2.3 Automated machine learning model construction

**Figure 1:**
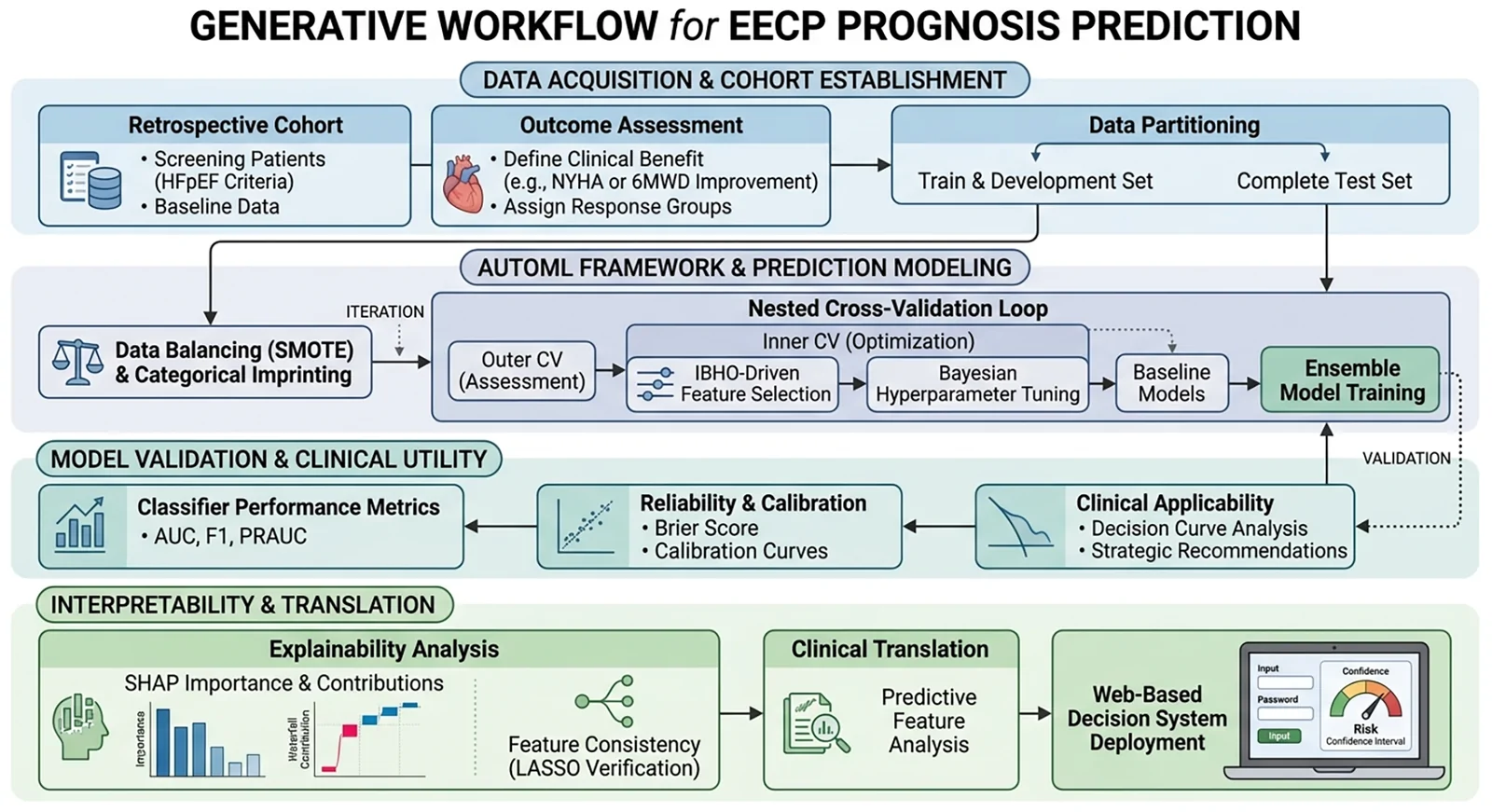
Technical roadmap.

To construct a prognostic prediction model without information leakage, this study designed and implemented a rigorous double-layered nested cross-validation AutoML framework, with the improved Newton's downhill optimizer (INDO) as its core. The technical roadmap of the study is shown in Figure 1.

#### 2.3.1 Algorithm selection and performance benchmarking

To identify a robust core optimization engine for the subsequent nested cross-validation AutoML framework, this study conducted performance benchmarking of the improved Newton's downhill optimizer (INDO) against seven mainstream algorithms. INDO was developed by introducing chaotic mapping for initial population distribution optimization and a dynamic Lévy flight step-size strategy to balance exploration and exploitation. The comparison included the whale optimization algorithm (WOA), grey wolf optimizer (GWO), particle swarm optimization (PSO), genetic algorithm (GA), and two hybrid algorithms (GA-PSO, GA-ACO). All benchmarking experiments were performed on the training-development set (*n* = 385). Progressive artificial perturbations (0%–10%, 10%–20%, 20%–30%, and 30%–40%) were injected to simulate measurement errors and missing values commonly encountered in real-world clinical data, and each algorithm was independently trained 30 times per perturbation level to calculate performance statistics. A feature selection module was also incorporated to evaluate model complexity control capability. This benchmarking phase was designed to provide quantitative evidence for algorithm selection prior to establishing the formal nested cross-validation modeling pipeline, rather than serving as a standalone algorithm validation study.

#### 2.3.2 Nested cross-validation modeling workflow

To ensure an unbiased evaluation of model performance, this study rigorously designed the entire modeling pipeline to strictly isolate the model development and final evaluation phases, ensuring that the independent test set remained completely untouched before the final model was finalized. The specific steps are as follows.

The first step was data partitioning. The overall dataset was randomly divided at a ratio of 7:3 into a training-development set (*n* = 385) and a one-time locked independent test set (*n* = 165). The training-development set was used for model development, feature screening, algorithm selection, and hyperparameter optimization; the independent test set did not participate in any model construction or parameter adjustment during the aforementioned processes and was only used for the final model performance evaluation.

The second step was the outer cross-validation. Five-fold cross-validation was adopted as the outer validation framework within the training-development set. In each outer iteration, the training-development set was further divided into an outer training fold and an outer validation fold, where the outer validation fold was used to simulate unseen data during the development phase to evaluate the stability and generalization performance of the modeling pipeline under different data splits.

The third step was the inner AutoML optimization. In each outer training fold, the INDO-driven automated machine learning optimization pipeline was executed. This inner optimization process completed the model configuration search based on internal cross-validation, with all data preprocessing, feature screening, model selection, and hyperparameter tuning confined to the inner training data. Specifically, preprocessing steps such as missing value imputation and variable standardization were first completed in the inner training sub-folds, followed by the application of the Synthetic Minority Oversampling Technique (SMOTE) for class balancing, and subsequently, the feature subset, candidate model types, and their hyperparameter configurations were jointly optimized through the INDO algorithm. All preprocessing rules, parameter estimates, feature selection results, and model hyperparameters were learned solely from the corresponding training sub-folds and applied to the corresponding validation sub-folds for performance calculation, thereby preventing information leakage across folds. At this stage, INDO simultaneously completed feature combination screening in the discrete space and hyperparameter search in the continuous space, with the optimization objective being the minimization of the preset objective function in the internal cross-validation.

The fourth step was outer model training and validation. For each outer iteration, the optimal feature subset, model algorithm, and hyperparameter configuration determined by the inner AutoML optimization were used to retrain the model on the corresponding outer training fold, and performance was evaluated on the outer validation fold, recording metrics such as ROC-AUC, PR-AUC, accuracy, sensitivity, specificity, F1 score, and Brier score for that fold. After completing five outer iterations, the results from the five outer validation folds were aggregated to evaluate the stability and generalization performance of the AutoML modeling pipeline within the training-development set.

The fifth step was final model determination. After completing the nested cross-validation assessment within the training-development set, the independent test set remained completely locked. Subsequently, based on the pre-specified candidate feature space, candidate algorithm space, hyperparameter search range, and optimization objective function, the INDO-AutoML optimization pipeline was re-executed on the entire training-development set (*n* = 385). This process automatically determined the final feature subset, final model algorithm, and optimal hyperparameter configuration through internal cross-validation within the training-development set. The finalized model configuration was not manually aggregated or selected by the researchers based on the median of the outer validation results, but was automatically searched by the predefined AutoML optimization pipeline on the complete training-development set. Subsequently, this final configuration was used to train on the entire training-development set to obtain the single fixed final prediction model.

The sixth step was independent test set evaluation. The finalized fixed AutoML prediction model described above was applied to the completely independent test set (*n* = 165), and performance metrics including ROC-AUC, PR-AUC, accuracy, sensitivity, specificity, F1 score, Brier score, calibration curve, and decision curve analysis were calculated and reported. Because the independent test set was not used in any of the processes of feature screening, model selection, hyperparameter optimization, and final model training, its evaluation results can reflect the generalization performance of the final model on unseen samples. For model comparison, this study simultaneously established five benchmark models—logistic regression (LR), support vector machine (SVM), adaptive boosting (AdaBoost), extreme gradient boosting (XGBoost), and light gradient boosting machine (LightGBM)—under the same data split and validation principles, and their performance was compared with that of the INDO-AutoML model.

### 2.4 Evaluation metrics

This study established a multidimensional comprehensive evaluation system to comprehensively assess the prediction model's discrimination, calibration, clinical utility, and the robustness of its results. All evaluations were strictly based on the independent locked test set to ensure unbiased performance estimates.

In terms of classification performance, the area under the receiver operating characteristic curve (ROC-AUC) and the area under the precision-recall curve (PR-AUC) were calculated as the primary evaluation metrics, with the latter being more sensitive to performance differences in class-imbalanced datasets. Meanwhile, based on a preset probability threshold of 0.5, accuracy (ACC), sensitivity (SEN), specificity (SPE), and F1 score were reported. To avoid optimistic bias introduced by threshold selection, all confusion matrix-based metrics were calculated using this default threshold, without *post-hoc* optimization on the test set. The positive outcome was defined as “EECP treatment effective.” In the overall cohort, the positive event rate was 60.00% (330/550). After random division at a 7:3 ratio, the positive rate in the training-development set was 58.70% (226/385), and the positive rate in the independent test set was 63.03% (104/165).

In terms of uncertainty quantification, to assess the variability of performance estimates due to limited sample size, this study calculated 95% confidence intervals (CIs) for all core metrics. The specific methods were as follows: For ROC-AUC and PR-AUC, 2000 stratified bootstrap resamples were used for estimation; for the Brier score, the confidence interval was derived after calculating its standard error based on the normal approximation method; for proportion-type metrics such as accuracy, sensitivity, specificity, and F1 score, the Clopper-Pearson exact method was used to calculate confidence intervals.

In terms of calibration assessment, the consistency between the model's predicted probabilities and the actual observed outcomes was examined from multiple perspectives. First, calibration curves were plotted, using locally weighted scatterplot smoothing (LOESS) to nonparametrically fit the relationship between the model's raw predicted probabilities and the actual outcomes, visually demonstrating the model's calibration trajectory. Second, the Brier score was calculated to quantify the overall mean squared error of the predicted probabilities. To further analyze the sources of calibration deviation, this study also decomposed and reported the calibration-in-the-large and the calibration slope. The calibration-in-the-large is the difference between the average predicted probability and the actual event rate in the test set, reflecting whether the model exhibits systematic overestimation or underestimation; the calibration slope was derived by fitting a logistic regression of the observed outcomes on the log odds of the predicted probabilities, with an ideal value of 1.0, and a significant deviation from 1.0 suggesting over-dispersion or under-calibration of predictions. It should be emphasized that no *post-hoc* calibration techniques were introduced throughout the entire modeling workflow, and all calibration assessments were based on the model's raw predicted probabilities to faithfully reflect its calibration performance.

In terms of clinical utility assessment, decision curve analysis (DCA) was used to quantify the net benefit of the prediction model in guiding clinical decision-making. The basic principle of DCA is to compare the net benefit generated by model-based intervention decisions against two default strategies — “treat all” (i.e., assuming all patients would benefit from EECP and providing treatment) and “treat none” (i.e., assuming no patients would benefit and not providing any EECP treatment) — at different risk thresholds (*p_t_*). The risk threshold *p_t_* here is defined as the probability cut-off point for “EECP treatment effective” predicted by the model; that is, when the model predicts a patient's probability of effective treatment is greater than or equal to *p_t_*, the patient is determined as positive and recommended to receive EECP treatment; this threshold reflects the clinical decision-maker's trade-off attitude between benefit and risk. The formula for calculating net benefit is NB = {TP−FP ×  [*p_t_*/(1−*p_t_*)]}/N, where *TP* is the number of true positives, *FP* is the number of false positives, and *N* is the total sample size of the evaluation set. The horizontal axis of the decision curve represents a continuous range of threshold probabilities, and the vertical axis represents the corresponding net benefit values. By observing in which threshold interval the model's net benefit curve lies above the two extreme strategy reference lines [the “treat all” line is a decreasing straight line from (*p_t_*, 0) to (1, 1−prevalence), and the “treat none” line is a horizontal line constant at zero], the applicable threshold range within which the model-aided decision-making yields positive clinical net benefit can be determined. This DCA was performed based on the independent test set, where the baseline event rate (i.e., the EECP treatment effectiveness rate) was 63.03% (104/165).

### 2.5 Interpretability analysis

After the preliminary screening of prognostic prediction features through the AutoML framework, LASSO regression analysis was further adopted to verify the robustness of the screened features, and finally, the clinical relevance of the features was interpreted using the SHAP model. The specific workflow was as follows: (1) AutoML preliminary feature screening: Based on a predefined search space and optimization objective, the AutoML algorithm was utilized to automatically identify the feature subset significantly associated with prognosis. (2) After the primary feature screening and final model construction under the AutoML framework, this study further adopted LASSO regression for supplementary consistency analysis to evaluate the stability of the core variables screened by AutoML within the traditional regularized regression framework. It should be emphasized that the LASSO analysis did not participate in the feature screening, algorithm selection, hyperparameter optimization, or final model construction of the AutoML model, and its results are not considered external validation evidence. This analysis only serves as an auxiliary reference for internal consistency and feature robustness, used to observe whether the variables selected by AutoML also exhibit a strong retention tendency in the sparse penalty model. The feature set of the final prediction model is still entirely determined by the preset INDO-AutoML optimization pipeline, rather than being replaced or expanded by the LASSO results. (3) SHAP (Shapley additive explanations) interpretability analysis: On this basis, this study further employed the SHAP method to conduct interpretability analysis on the final AutoML model, so as to quantify the contribution direction and contribution strength of each core variable to the individualized prediction results. The analysis of model logic strictly followed the SHAP framework based on coalitional game theory. Numerical attribution of feature contributions to individual predictions was performed by calculating Shapley values. Summary plots were comprehensively used to reveal the global feature importance ranking, and the interpretation process of specific predictions was intuitively presented through waterfall plots, decision path plots, and force plots.

### 2.6 Decision support system development

Based on the finalized AutoML prognostic model validated on the test set, a clinically-oriented decision support tool (web-based risk calculator) was further developed. This system integrates the constructed machine learning prediction module, takes the key variables screened by the model as input, synchronously outputs individualized risk estimates for EECP treatment outcomes in HFpEF patients, and generates actionable follow-up recommendations in combination with the variable profile, thereby providing the medical team with an intuitive and low-threshold auxiliary decision-making reference.

### 2.7 Statistical methods

The study data were uniformly imported into the SPSS 26.0 statistical analysis platform for standardized processing. Continuous variables conforming to a normal distribution were expressed as mean ± standard deviation (x¯ ± s), continuous variables not conforming to a normal distribution were expressed as M [Q1, Q3], and categorical variables were expressed as frequencies and percentages [n (%)]. For intergroup comparisons, the normality of continuous variables was first assessed using the Shapiro–Wilk test. If both groups of data conformed to a normal distribution (Shapiro–Wilk *P* > 0.05), the independent-samples t-test was used for intergroup comparison; if the data did not conform to a normal distribution, the Mann–Whitney U test was used. For categorical variables, intergroup comparison was performed using the Pearson chi-square test or Fisher's exact test, as appropriate based on expected frequencies. Prior to formal analysis, the distributional characteristics of all continuous variables were reviewed for potential outliers. No observations meeting the statistical definition of an outlier requiring special handling were identified. The test power was based on the *P* value (two-sided test, with the significance threshold set at *α*=0.05), and the study results were presented in a structured tabular format.

## 3 Results

### 3.1 Characteristics of study population

This study included a total of 550 HFpEF patients, with a mean age of 67.63 ± 8.22 years, including 246 males (44.73%) and 304 females (55.27%). Except for a marginal difference in age (training set: 67.17 ± 8.20 years vs. test set: 68.70 ± 8.18 years, *P* = 0.045), no statistically significant differences were observed between the training and test sets in all other baseline characteristics (all *P* > 0.05), indicating that the random allocation yielded largely comparable groups, indicating successful random allocation and good comparability between the two groups, as detailed in Table 1. The overall EECP treatment effectiveness rate was 60.00% (330/550). Significant differences were observed between the effective group and the ineffective group in multiple baseline characteristics. Patients in the effective group were older (69.71 ± 7.84 years vs. 64.51 ± 7.79 years, *P* < 0.001), had lower body mass index (27.74 ± 3.82 kg/m^2^ vs. 29.28 ± 4.42 kg/m^2^, *P* < 0.001), longer 6-minute walk distance (347.68 ± 69.77 meters vs. 287.65 ± 76.15 meters, *P* < 0.001), higher left ventricular ejection fraction (59.25 ± 4.22% vs. 56.43 ± 3.73%, *P* < 0.001), lower left atrial volume index (35.90 ± 9.02 mL/m^2^ vs. 41.62 ± 10.12 mL/m^2^, *P* < 0.001), and lower E/e' ratio (11.85 ± 3.37 vs. 13.20 ± 3.34, *P* < 0.001). No significant differences were found between the two groups in N-terminal pro-B-type natriuretic peptide levels, duration of heart failure, and tricuspid regurgitation velocity, as detailed in Table 2.

**Table 1:** Comparison of baseline characteristics between the training Set and the test Set.

Variable	Training_set (*n* = 385)	Test_set (*n* = 165)	Test statistic	*P* value
Outcome variable			*χ*^2^ = 0.902	0.342
• EECP effective, n (%)	226 (58.70%)	104 (63.03%)		
• EECP ineffective, n (%)	159 (41.30%)	61 (36.97%)		
Age, mean ± SD years	67.17 ± 8.20	68.70 ± 8.18	t = −2.008	0.045
BMI, mean ± SD kg/m^2^	28.35 ± 4.08	28.36 ± 4.29	t = −0.021	0.983
HF_duration, mean ± SD years	4.30 ± 2.69	4.23 ± 2.48	t = 0.263	0.793
NT_proBNP, mean ± SD pg/mL	854.36 ± 468.43	854.50 ± 396.75	t = −0.003	0.997
6MWD, mean ± SD meters	322.29 ± 78.29	326.90 ± 77.74	t = −0.635	0.526
LVEF, mean ± SD %	58.20 ± 4.36	57.95 ± 4.01	t = 0.642	0.521
LVMI, mean ± SD g/m^2^	115.27 ± 23.99	111.91 ± 27.71	t = 1.434	0.152
LAVI, mean ± SD mL/m^2^	38.05 ± 9.91	38.52 ± 9.80	t = −0.516	0.606
E_e_ratio, mean ± SD	12.42 ± 3.53	12.31 ± 3.13	t = 0.354	0.724
TR_velocity, mean ± SD m/s	2.80 ± 0.49	2.79 ± 0.47	t = 0.068	0.946
EECP_sessions, mean ± SD sessions	35.22 ± 4.84	35.43 ± 5.36	t = −0.445	0.657
EECP_duration, mean ± SD weeks	7.06 ± 1.04	7.02 ± 1.03	t = 0.394	0.693
Sex, n (%)			χ^2^ = 2.964	0.085
• Male	163 (42.34%)	83 (50.30%)		
• Female	222 (57.66%)	82 (49.70%)		
Smoking_status, n (%)			χ^2^ = 1.950	0.377
• Never	198 (51.43%)	86 (52.12%)		
• Former	126 (32.73%)	60 (36.36%)		
• Current	61 (15.84%)	19 (11.52%)		
Diabetes, n (%)			χ^2^ = 0.027	0.869
• No	275 (71.43%)	119 (72.12%)		
• Yes	110 (28.57%)	46 (27.88%)		
Hypertension, n (%)			χ^2^ = 0.495	0.482
• No	157 (40.78%)	62 (37.58%)		
• Yes	228 (59.22%)	103 (62.42%)		
NYHA_class, n (%)			χ^2^ = 2.194	0.334
• II	161 (41.82%)	77 (46.67%)		
• III	203 (52.73%)	83 (50.30%)		
• IV	21 (5.45%)	5 (3.03%)		
ACEI_ARB, n (%)			χ^2^ = 3.415	0.065
• No	146 (37.92%)	49 (29.70%)		
• Yes	239 (62.08%)	116 (70.30%)		
Beta_blocker, n (%)			χ^2^ = 0.975	0.324
• No	92 (23.90%)	46 (27.88%)		
• Yes	293 (76.10%)	119 (72.12%)		
MRA, n (%)			χ^2^ = 0.546	0.460
• No	225 (58.44%)	102 (61.82%)		
• Yes	160 (41.56%)	63 (38.18%)		
SGLT2i, n (%)			χ^2^ = 0.011	0.918
• No	276 (71.69%)	119 (72.12%)		
• Yes	109 (28.31%)	46 (27.88%)		

**Table 2:** Comparison of baseline characteristics stratified by outcome.

Variable	Effective_group (*n* = 330)	Ineffective_group (*n* = 220)	Test statistic	*P* value
Age, mean ± SD years	69.71 ± 7.84	64.51 ± 7.79	t = 7.643	<0.001
BMI, mean ± SD kg/m^2^	27.74 ± 3.82	29.28 ± 4.42	t = −4.338	<0.001
HF_duration, mean ± SD years	4.39 ± 2.67	4.11 ± 2.56	t = 1.236	0.217
NT_proBNP, mean ± SD pg/mL	848.90 ± 444.94	862.67 ± 452.89	t = −0.353	0.724
6MWD, mean ± SD meters	347.68 ± 69.77	287.65 ± 76.15	t = 9.528	<0.001
LVEF, mean ± SD %	59.25 ± 4.22	56.43 ± 3.73	t = 8.062	<0.001
LVMI, mean ± SD g/m^2^	113.15 ± 24.86	115.93 ± 25.64	t = −1.268	0.205
LAVI, mean ± SD mL/m^2^	35.90 ± 9.02	41.62 ± 10.12	t = −6.942	<0.001
E_e_ratio, mean ± SD	11.85 ± 3.37	13.20 ± 3.34	t = −4.611	<0.001
TR_velocity, mean ± SD m/s	2.77 ± 0.50	2.83 ± 0.46	t = −1.422	0.156
EECP_sessions, mean ± SD sessions	35.52 ± 4.95	34.94 ± 5.06	t = 1.321	0.187
EECP_duration, mean ± SD weeks	7.05 ± 1.01	7.06 ± 1.08	t = −0.151	0.88
Sex, n (%)			χ^2^ = 3.443	0.064
• Male	137 (41.52%)	109 (49.55%)		
• Female	193 (58.48%)	111 (50.45%)		
Smoking_status, n (%)			χ^2^ = 0.555	0.758
• Never	169 (51.21%)	115 (52.27%)		
• Former	110 (33.33%)	76 (34.55%)		
• Current	51 (15.45%)	29 (13.18%)		
Diabetes, n (%)			χ^2^ = 4.189	0.041
• No	247 (74.85%)	147 (66.82%)		
• Yes	83 (25.15%)	73 (33.18%)		
Hypertension, n (%)			χ^2^ = 0.182	0.67
• No	129 (39.09%)	90 (40.91%)		
• Yes	201 (60.91%)	130 (59.09%)		
NYHA_class, n (%)			χ^2^ = 3.200	0.202
• II	152 (46.06%)	86 (39.09%)		
• III	165 (50.00%)	121 (55.00%)		
• IV	13 (3.94%)	13 (5.91%)		
ACEI_ARB, n (%)			χ^2^ = 1.192	0.275
• No	111 (33.64%)	84 (38.18%)		
• Yes	219 (66.36%)	136 (61.82%)		
Beta_blocker, n (%)			χ^2^ = 0.929	0.335
• No	78 (23.64%)	60 (27.27%)		
• Yes	252 (76.36%)	160 (72.73%)		
MRA, n (%)			χ^2^ = 0.454	0.501
• No	200 (60.61%)	127 (57.73%)		
• Yes	130 (39.39%)	93 (42.27%)		
SGLT2i, n (%)			χ^2^ = 3.743	0.053
• No	227 (68.79%)	168 (76.36%)		
• Yes	103 (31.21%)	52 (23.64%)		

### 3.2 Algorithm selection and robustness benchmarking

**Figure 2:**
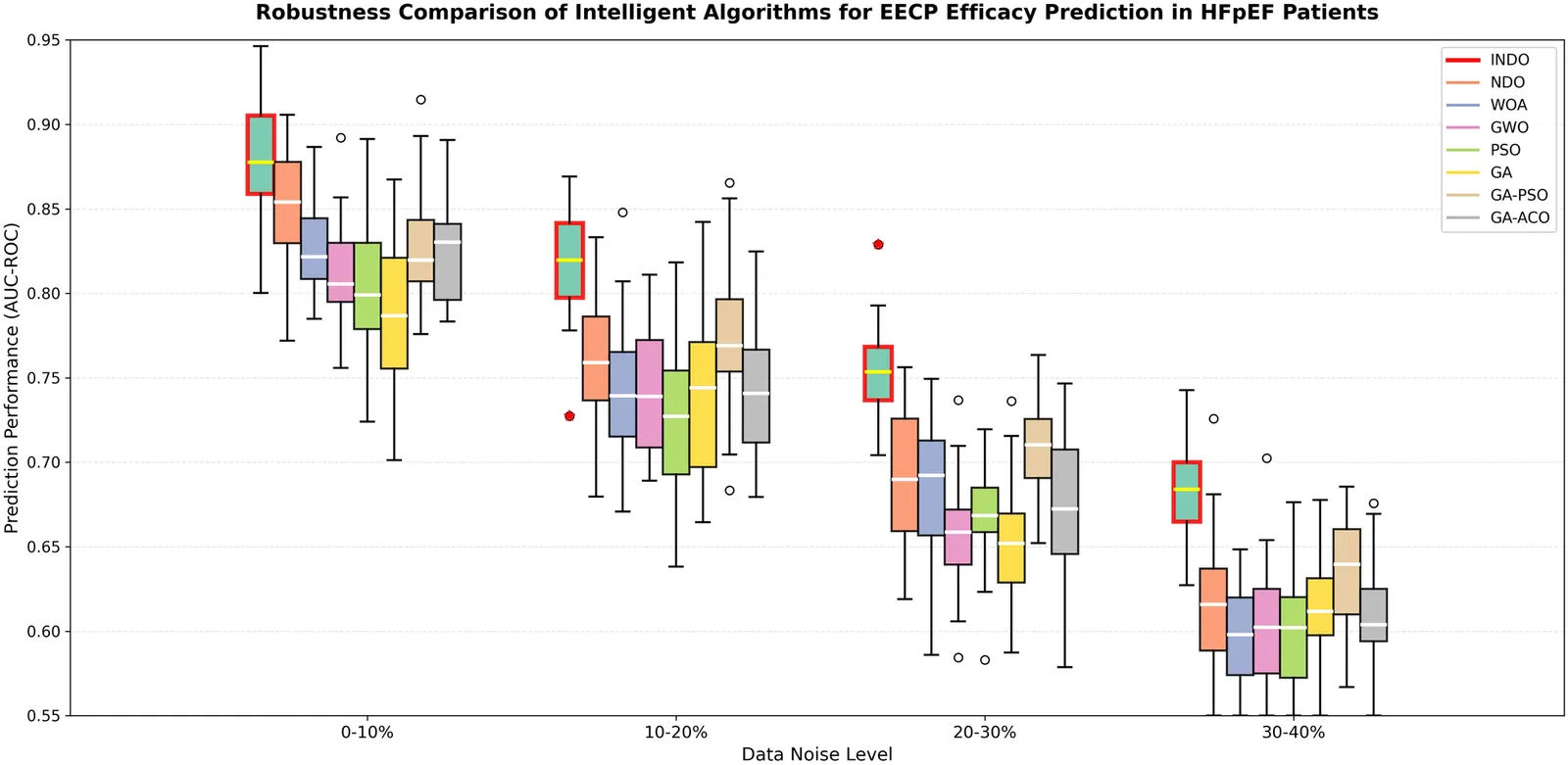
Comparison of the robustness of intelligent algorithms in predicting composite postoperative outcomes. This figure illustrates the stability performance of eight intelligent algorithms under increasing data perturbations. The box plots represent the distribution of cross-validated AUC values from 30 replicate experiments.

During the algorithm selection and performance benchmarking phase, this study compared the stability performance of INDO against seven mainstream optimization algorithms on the training-development set under data quality perturbations, providing empirical evidence for algorithm selection in the final AutoML modeling pipeline. As perturbation intensity increased, all algorithms exhibited performance decline, with the average AUC decreasing from 0.826 under low perturbation (0%–10%) to 0.620 under high perturbation (30%–40%), representing a relative decline of 24.9%. INDO demonstrated the best stability, with its AUC declining from 0.880 to 0.683 (relative decline rate 22.4%), significantly outperforming the NDO group (decline rate 27.8%) and maintaining the highest absolute accuracy under high perturbation conditions (Figure 2).

**Figure 3:**
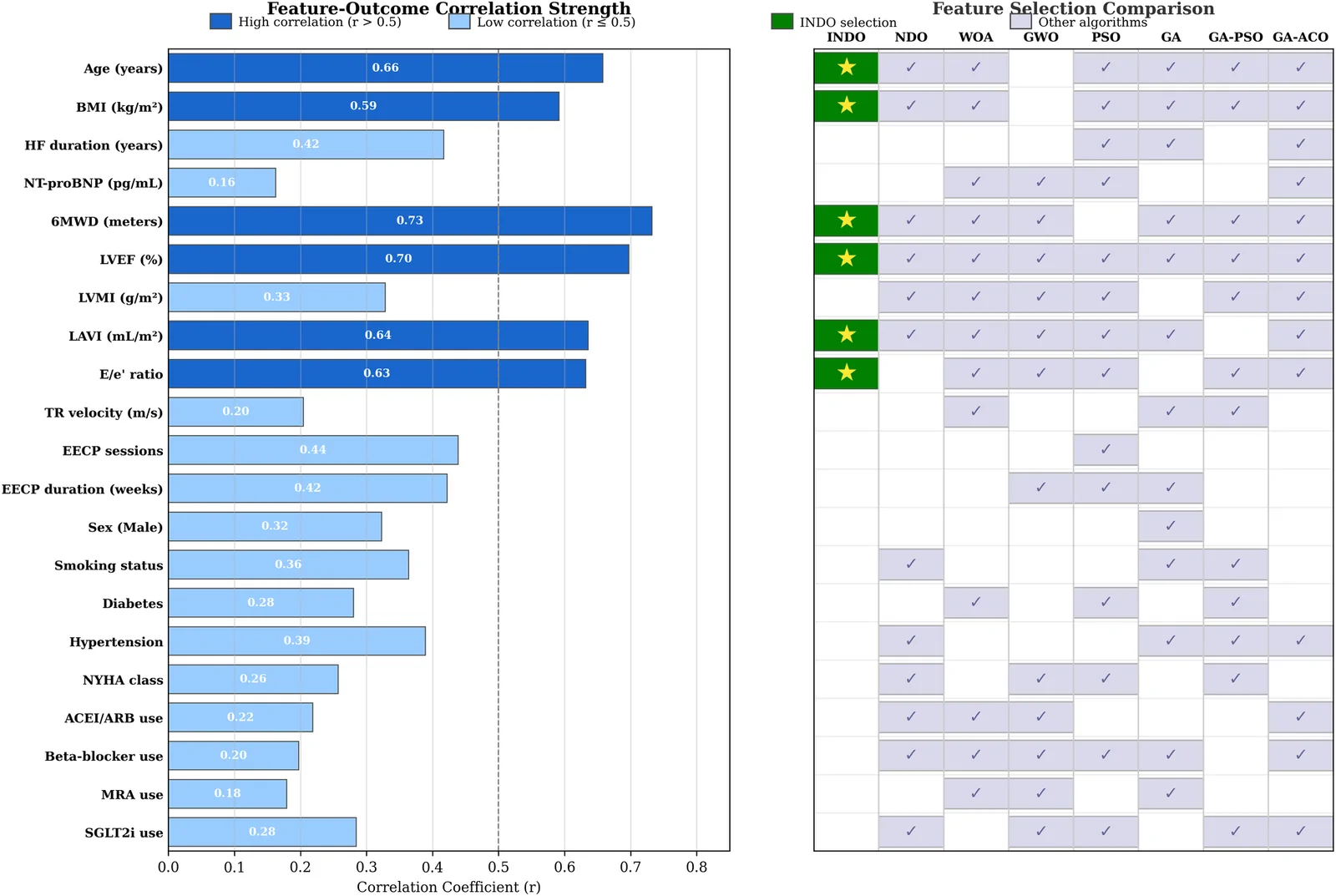
Clinical feature analysis and feature selection optimization. This study compared the correlation strength between 15 clinical features and prognostic outcomes, and evaluated the feature selection performance of the INDO algorithm versus other optimization algorithms (NDO, WOA, GWO, PSO, GA, GA-PSO, GA-ACO). The left panel shows the correlation coefficient (r) between each feature and the outcome, with dark blue indicating highly correlated features (r > 0.5), light blue indicating weakly correlated features (r ≤ 0.5), and the gray dashed line representing the correlation threshold of 0.5. The right panel shows the feature selection results of the algorithms, where dark green squares indicate features selected by INDO and light blue squares indicate features selected by other algorithms.

In key feature identification, INDO stably screened 6 highly relevant predictors, representing a 52.8% reduction compared with the average of 12.7 features selected by the other seven algorithms, effectively reducing model complexity while maintaining prediction accuracy (Figure 3).

### 3.3 Model training

**Figure 4:**
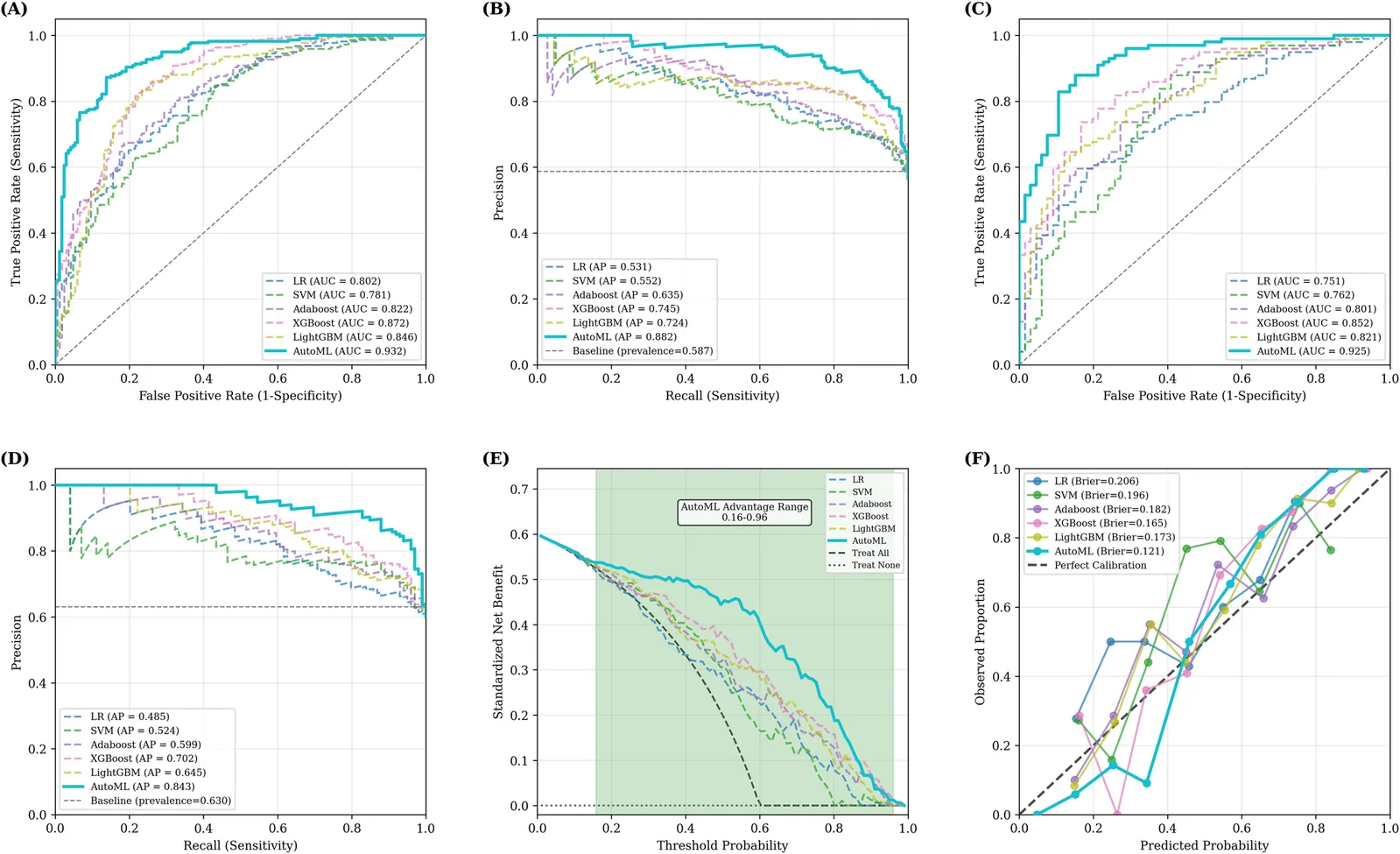
Training and performance validation of predictive models for the primary outcome. **(A)** ROC curves on the training set; **(B)** PR curves on the training set; **(C)** ROC curves on the test set; **(D)** PR curves on the test set; **(E)** DCA curve on the test set; **(F)** Calibration curve on the test set.

This study evaluated the predictive performance of six machine learning models on the training set. The results showed that the AutoML model exhibited optimal performance, with a ROC-AUC of 0.9323 and a PR-AUC of 0.8821 (Table 3, Figures 4C,D). Notably, the advantage of AutoML in the F1 score (0.8473) was particularly prominent, indicating its stronger clinical application value in balancing precision and recall. The features ultimately screened by AutoML were: 6-minute walk distance (6MWD), left ventricular ejection fraction (LVEF), age, left atrial volume index (LAVI), E/e' ratio, and body mass index (BMI).

**Table 3:** Performance evaluation metrics of predictive models.

Model	Data set	PRE	SEN	SPE	ACC	F1	ROC-AUC	PR-AUC
LR	Training set	0.5521	0.7025	0.7824	0.7589	0.6183	0.8023	0.5312
	Test set	0.531	0.6125	0.7953	0.7325	0.5689	0.7512	0.4854
SVM	Training set	0.5128	0.7236	0.7215	0.7228	0.6012	0.7815	0.5523
	Test set	0.5012	0.6875	0.7321	0.7189	0.5798	0.7623	0.5236
Adaboost	Training set	0.6425	0.6528	0.8623	0.8023	0.6475	0.8215	0.6352
	Test set	0.5987	0.7125	0.8236	0.7854	0.6512	0.8012	0.5987
XGBoost	Training set	0.7321	0.7125	0.8956	0.8452	0.7221	0.8723	0.7452
	Test set	0.7012	0.7625	0.8654	0.8321	0.7305	0.8521	0.7023
LightGBM	Training set	0.6823	0.7321	0.8523	0.8125	0.7054	0.8456	0.7236
	Test set	0.6323	0.7125	0.8036	0.7712	0.6703	0.8212	0.6454
AutoML	Training set	0.8525	0.8425	0.9425	0.9123	0.8473	0.9323	0.8821
	Test set	0.8154	0.8425	0.9156	0.8954	0.8285	0.9254	0.8425

Evaluating the final performance of the model on the completely locked independent test set, the results showed that the AutoML model demonstrated excellent and predictive performance (Figures 4C,D). Its discrimination metrics reached a high level, with a ROC-AUC of 0.925 (95% CI, 0.883–0.952) and a PR-AUC of 0.843 (95% CI, 0.768–0.901), both significantly outperforming all benchmark models. At the default probability threshold of 0.5, the model exhibited balanced classification performance, with an accuracy of 0.895, an F1 score of 0.829 (95% CI, 0.771–0.870), a sensitivity of 0.843 (95% CI, 0.758–0.902), and a specificity of 0.916 (95% CI, 0.825–0.962).

Decision curve analysis (Figure 4E) showed that applying the AutoML predictive model within a risk threshold range of 16% to 96% on the test set yielded greater clinical net benefit compared with traditional methods.

In terms of probability calibration, the Brier score of the AutoML model on the test set was 0.121 (95% CI, 0.082–0.159), indicating a very low overall probability error. Further decomposition of calibration revealed a calibration-in-the-large of −0.05, suggesting that the average predicted probability of the model only slightly underestimated the true effectiveness rate; the calibration slope was 1.06, which was extremely close to the ideal value of 1.0, indicating no systematic problems of over-dispersion or under-calibration in the risk distribution predicted by the model. The calibration curve plot (Figure 4F) visually demonstrated a high degree of fit between the LOESS-smoothed line and the ideal diagonal line, supporting the good calibration characteristics revealed by the aforementioned quantitative metrics.

### 3.4 Analysis of key influencing factors

#### 3.4.1 LASSO

**Figure 5:**
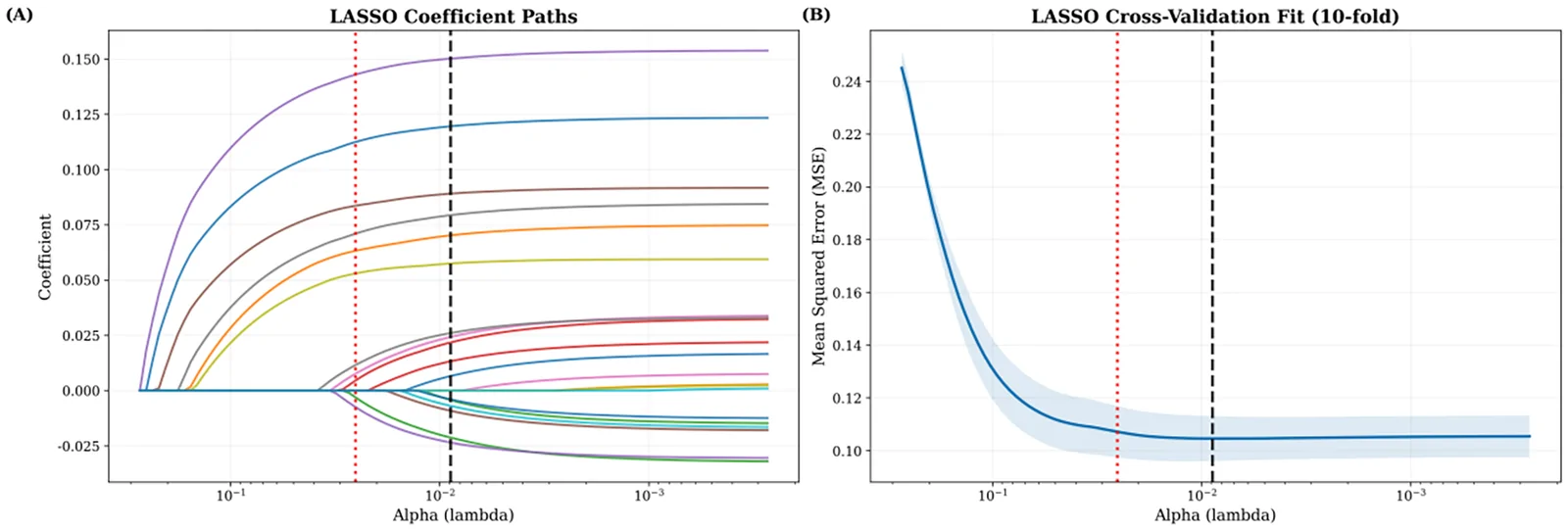
LASSO regression results. **(A)** LASSO trajectory plot; **(B)** LASSO cross-validation fitting plot.

To evaluate the consistency of features screened by AutoML within the traditional regularized regression framework, this study conducted a supplementary LASSO regression analysis on the training-development set (Figure 5). Under the Lambda1SE criterion, LASSO screened out several candidate variables associated with EECP treatment efficacy, including age, body mass index, 6-minute walk distance (6MWD), left ventricular ejection fraction (LVEF%), left atrial volume index (LAVI), and E/e' ratio, covering the six core predictors included in the final AutoML model. This result suggests that the main variables screened by the AutoML model also exhibit good consistency under the sparse penalty regression framework.

It should be noted that the purpose of the LASSO analysis was neither to redefine the final model feature set nor to constitute an independent external validation. Although the additional variables screened by LASSO may have some statistical association with EECP treatment efficacy, they failed to achieve a better balance among feature subset compactness, overall model performance, cross-validation stability, and overfitting control during the nested optimization process of INDO-AutoML. Therefore, the final model retains the six core variables determined by the AutoML pipeline to ensure model parsimony, clinical interpretability, and deployment convenience.

#### 3.4.2 SHAP analysis

**Figure 6:**
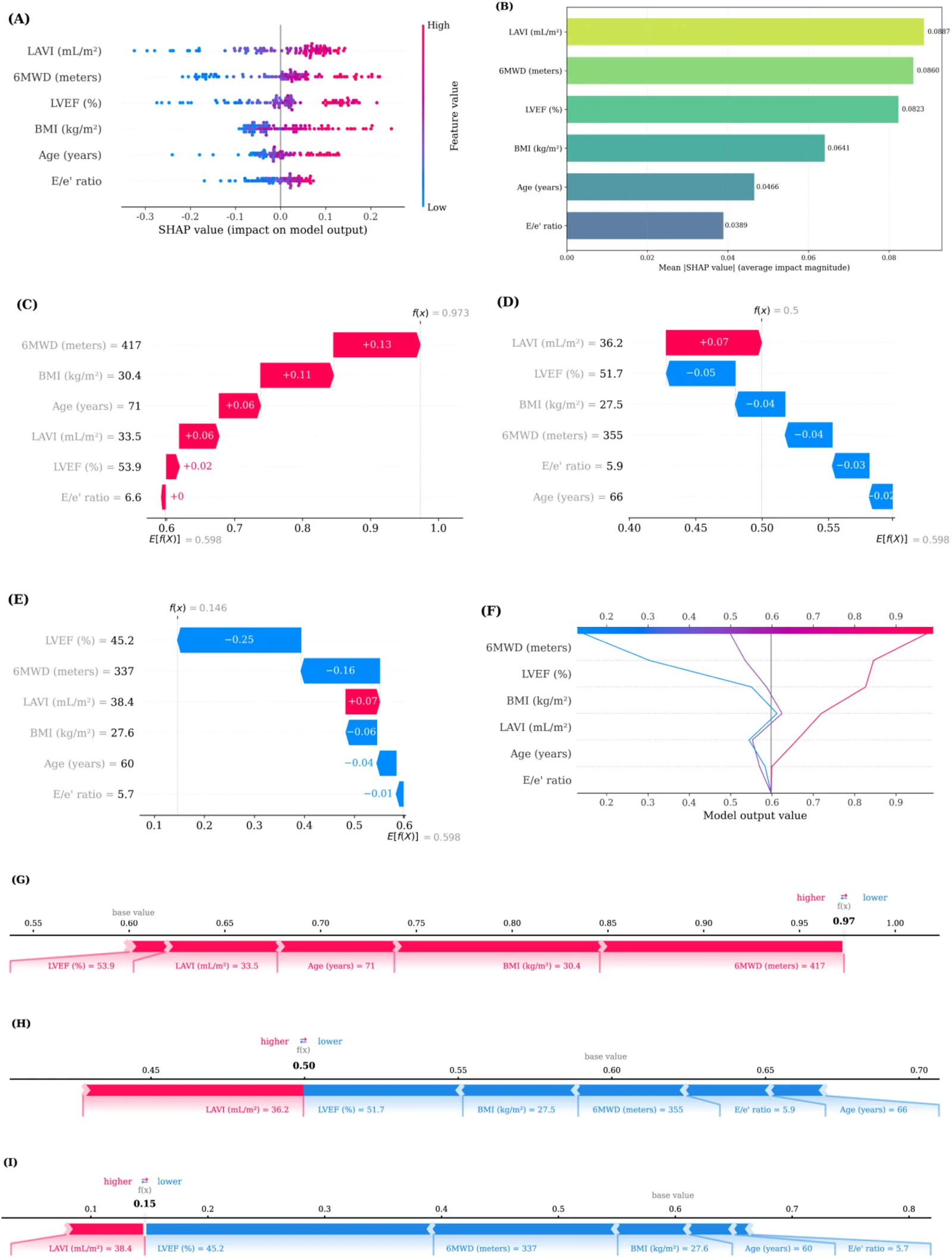
SHAP interpretability analysis. **(A)** SHAP summary plot; **(B)** SHAP feature importance plot; **(C**, **D**, and **E)** Waterfall plots illustrating the cumulative contribution process of each feature to the prediction of individual patients. The baseline value represents the model's average prediction for all patients, while the feature contributions show how each feature influences the final prediction (red indicates increased risk, blue indicates decreased risk). The sum of all feature contributions yields the final predicted value; **(F)** Decision path plot comparing the decision paths of multiple patients, showing how different feature combinations lead to different prediction outcomes. The horizontal axis shows the predicted probability, the vertical axis lists the features, and the curving paths trace the decision routes from baseline value to final prediction; **(G**, **H**, and **I)** Force plots intuitively demonstrate how each feature pushes the prediction towards higher or lower risk. Red arrows indicate features pushing the prediction towards higher risk, blue arrows indicate features pushing towards lower risk, and the arrow length indicates the magnitude of influence.

The variable importance assessment based on SHAP (Shapley Additive Explanations) analysis (Figures 6A,B) showed that the main factors influencing the efficacy of EECP treatment in HFpEF patients, in order of importance, were 6-minute walk distance (6MWD), left ventricular ejection fraction (LVEF), age (Age), left atrial volume index (LAVI), E/e' ratio, and body mass index (BMI). This ranking reflects the key influence of disease severity, cardiac functional status, and patient baseline characteristics on treatment outcomes. Through decision path analysis (Figure 6F), significant feature differences were observed among patients with different predicted outcomes, indicating good clinical interpretability of the model's predictions.

Further individualized analysis revealed typical feature patterns for different outcome prediction levels:

High treatment response prediction group (Figures 6C,G): Patients in this subgroup were older (71 years), but had a longer 6MWD (417 meters), LVEF maintained within a relatively ideal range (53.9%), and a lower E/e' ratio (6.6), suggesting relatively preserved left ventricular diastolic function, while the left atrium was not significantly enlarged (LAVI 33.5 mL/m^2^). Although BMI was high (30.4 kg/m^2^), other parameters indicated that this group might benefit from the circulatory improvement effects of EECP.

Moderate treatment response prediction group (Figures 6D,H): This patient (66 years) had lower 6MWD (355 meters) and LVEF (36.3%) than the high response group, with the E/e' ratio (5.9) and LAVI (36.2 mL/m^2^) suggesting mild diastolic dysfunction, and moderate BMI (27.5 kg/m^2^), indicating that their response to EECP might be partially limited.

Low treatment response prediction group (Figures 6E,I): This patient was 60 years old. Although younger, they had poor exercise tolerance (6MWD 337 meters), LVEF (45.2%) still at the lower limit of the normal range, and elevated LAVI (38.4 mL/m^2^), possibly suggesting aggravated left atrial remodeling, with high BMI (27.6 kg/m^2^). Although the E/e' ratio (5.7) was acceptable, the elevated LAVI might be a key factor limiting treatment benefit.

These findings suggest that 6MWD, LVEF, and LAVI contribute to predicting EECP efficacy, and that age and BMI may modulate treatment response through their effects on exercise tolerance and hemodynamics. The model may assist in identifying patients who could benefit and offers predictive information for individualized decision-making.

### 3.5 Clinical decision support system development

**Figure 7:**
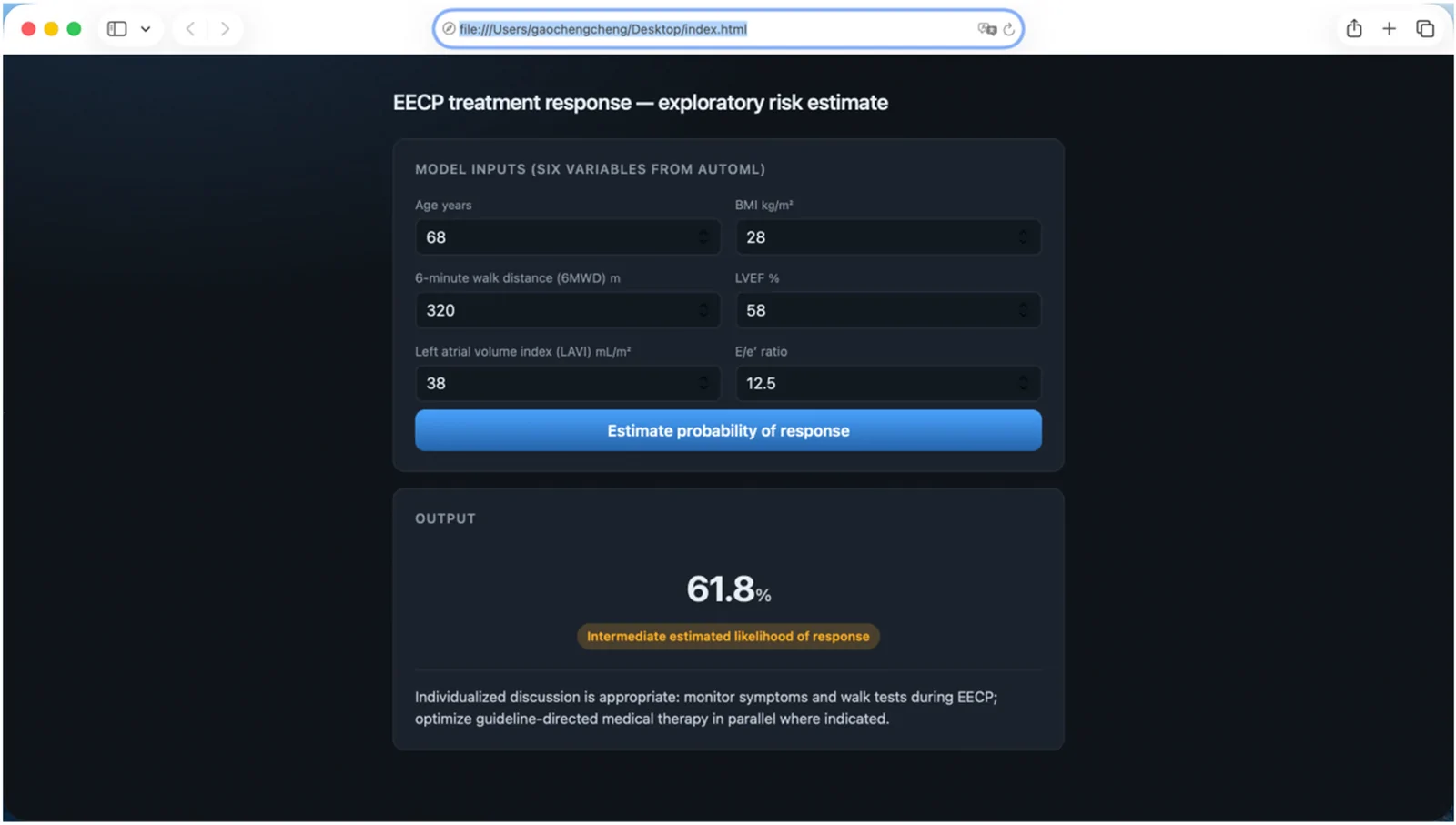
Software system demonstration.

The aforementioned results have successfully identified the key features influencing EECP treatment outcomes in HFpEF patients. However, in actual clinical application, the changes in these features are complex and intricate, making it difficult to intuitively reveal patient treatment outcomes. Existing artificial intelligence methods have relatively high barriers to promotion and application, requiring clinical staff to possess high programming skills and extensive literature knowledge, which makes them difficult to promote and use in the vast majority of hospitals. To address this issue, this study innovatively constructed a practical visualization system built on the basis of the selected key features, possessing the application advantages of intuitiveness, convenience, and practicality. During the application of the visualization system, users only need to input the specific numerical values of the six key features in the “Feature Input” column, and the system will automatically calculate the probability of EECP treatment outcomes in HFpEF patients and provide clinical recommendations, as shown in Figure 7.

## 4 Discussion

This study, based on a retrospective cohort study design, integrated baseline clinical characteristics and multi-dimensional echocardiographic parameters of HFpEF patients, successfully constructing and validating an AutoML prognostic prediction model driven by an improved Newton's downhill optimizer for assessing individualized efficacy of EECP treatment. The study results showed that, compared with traditional logistic regression, support vector machines, and various ensemble learning models, the AutoML model optimized based on INDO demonstrated significant advantages in core dimensions including discrimination, calibration, and clinical net benefit, confirming the feasibility and application potential of the AutoML framework in the clinical proposition of predicting EECP efficacy in HFpEF patients. Under the rigorous guarantee of a double-layered nested cross-validation framework, the prediction model constructed in this study not only achieved classification performance on the internal test set, but also revealed key predictive factors influencing treatment response through SHAP interpretability analysis, providing the necessary evidence-based foundation and biological plausibility support for the translation of this model from algorithm construction to clinical decision support.

The primary finding of this study is that the INDO algorithm-driven AutoML framework significantly outperformed traditional statistical models and mainstream ensemble learning models in predicting EECP treatment efficacy. At the algorithm selection level, prior to establishing the nested cross-validation framework, this study conducted systematic robustness benchmarking of INDO on the training-development set. The results demonstrated that under high-perturbation conditions, the relative decline in prediction accuracy for INDO was substantially lower than that of other algorithms, and its screened feature subsets were more compact (averaging only 6 variables, representing a 52.8% reduction compared with other algorithms), effectively reducing model complexity while maintaining predictive accuracy. This algorithmic superiority translated into dual benefits for the subsequent AutoML modeling pipeline: first, it ensured the stability of the optimization process under data quality fluctuations, preventing the inner-loop AutoML hyperparameter search from being trapped in local optima due to noisy data; second, the compactness of the feature subsets effectively enhanced the generalization capacity and clinical deployability of the final predictive model, mitigating overfitting risks associated with redundant features. Thus, INDO serves not merely as an independent optimization tool but as a core foundation ensuring the stable operation of the entire AutoML pipeline and the performance of the final model. At the methodological level, the core advantage of AutoML lies in its ability to adaptively complete the coordinated optimization of feature subset selection, model algorithm selection, and hyperparameter configuration within a pre-defined joint search space through algorithm-driven search strategies, thereby approximating the globally optimal model configuration under limited computational resources. Traditional manual feature engineering and preset model architectures rely heavily on researchers' prior experience, making it difficult to guarantee obtaining the optimal solution in the full candidate feature space, whereas the AutoML framework effectively overcomes this methodological limitation by automating the integration of key decision-making steps in the modeling process. Meanwhile, this study strictly implemented a double-layered nested cross-validation strategy during the model training process, ensuring that feature selection, hyperparameter tuning, and model evaluation were all completed independently on different data subsets, fundamentally avoiding the risk of information leakage and providing a solid methodological guarantee for the unbiased estimation of model performance metrics. In comparison with previous studies (19–21), although scholars have applied machine learning methods to the prognostic assessment of heart failure patients, most research has been limited to simple comparisons of single algorithms and preset feature sets, lacking systematic optimization of model selection and feature engineering strategies. This study, by introducing the AutoML framework into the field of EECP efficacy prediction for HFpEF patients, fills the methodological gaps of existing research and provides a more rigorous and efficient modeling paradigm for subsequent related studies.

At the level of correlation analysis between predictive factors and treatment efficacy, the baseline characteristic comparison results of this study showed that significant differences existed between the treatment-effective group and the ineffective group in multiple clinical indicators and echocardiographic parameters, and these differences provide important clues for understanding the heterogeneity of EECP treatment response. From the perspective of demographic characteristics, the mean age of patients in the effective group was significantly higher than that of the ineffective group, while body mass index was significantly lower. The phenomenon that older patients are more likely to benefit from EECP treatment may be related to the longer disease course of diastolic dysfunction and more severe vascular endothelial impairment in elderly HFpEF patients. The hemodynamic benefits brought by EECP through improving vascular endothelial function and promoting collateral circulation establishment may be more pronounced in this patient population (22, 23). Conversely, the relatively poorer efficacy in patients with higher body mass index may be related to obesity-related systemic inflammatory status, microvascular dysfunction, and increased comorbidity burden attenuating the therapeutic effects of EECP (24). In the assessment of multi-dimensional echocardiographic parameters, the effective group had higher LVEF levels, while left atrial volume index and E/e' ratio were significantly lower than those in the ineffective group. Although LVEF is generally maintained within the normal range in HFpEF patients, those at the lower limit of normal may have a degree of subclinical systolic dysfunction, which could affect the compensatory increase in cardiac output during EECP treatment (25). Left atrial volume index, as an important indicator reflecting the cumulative effect of chronic left ventricular diastolic dysfunction, marks more severe diastolic dysfunction and atrial remodeling with its elevation. The significantly increased left atrial volume index in the ineffective group suggests that severe atrial remodeling may constitute the pathophysiological basis limiting the hemodynamic benefits of EECP (26). The E/e' ratio is a core non-invasive parameter for assessing left ventricular filling pressure; the significantly higher E/e' ratio in the ineffective group compared to the effective group indicates that excessively elevated left ventricular filling pressure may attenuate the efficacy of EECP in increasing coronary perfusion during diastole, thereby limiting therapeutic benefits (27). It is noteworthy that tricuspid regurgitation velocity did not show a statistically significant difference between the two groups, which may suggest that the weight of right heart-pulmonary artery coupling function in predicting EECP efficacy is relatively limited, and its predictive value requires further validation in prospective cohorts with larger sample sizes. Furthermore, the difference in N-terminal pro-B-type natriuretic peptide levels between the two groups did not reach statistical significance, which differs to some extent from the conclusions of some previous studies (28, 29). The potential explanation for this phenomenon is that the HFpEF patients included in this study were all in a relatively stable compensated state, with a concentrated distribution range of N-terminal pro-B-type natriuretic peptide. Moreover, its level was affected by multiple confounding factors such as age, renal function, and atrial fibrillation, thus failing to fully demonstrate its predictive value in univariate comparison.

This study strictly followed the standardized process for the construction of prognostic prediction models in its design and implementation, but several limitations remain to be addressed in future research. First, this study adopted a two-center retrospective cohort study design. Although internal validity was ensured through strict inclusion and exclusion criteria and complete follow-up data, the geographical limitations and patient population selection bias inherent in two-center data inevitably constrain the generalizability of the model. Differences in EECP treatment protocol parameters, procedural standardization levels, and patient baseline characteristics may exist across different medical centers, all of which may affect the predictive performance of the model on external datasets. Although the AutoML model demonstrated performance on the internal independent test set, the current study lacks an external validation dataset from independent medical centers. This represents a significant limitation for the clinical deployment of the proposed decision-support web tool. Notably, a multi-center external validation study has already been initiated by our group to evaluate the model's portability and generalizability across different clinical settings and patient populations, and the results will be reported in a subsequent publication. Therefore, multi-center external validation studies are needed in the future to evaluate the model's portability and robustness across different clinical scenarios and population characteristics. Second, although the definition of EECP treatment efficacy in this study was based on objective assessment criteria of cardiac function improvement and exercise tolerance enhancement, as a binary outcome variable, the setting of its cut-off value may influence the distribution of the model's positive event rate. Furthermore, the composite definition of the primary outcome, combining NYHA class improvement and 6MWD increase with an “either-or” rule, may result in outcome misclassification if either component is measured with error. To mitigate this concern, NYHA class was assessed by blinded cardiologists using a standardized protocol, and the 6MWD was measured according to the American Thoracic Society guidelines. Finally, while the ≥30 m absolute threshold for 6MWD is well-established as the MCID in heart failure populations, we acknowledge that it does not account for baseline exercise capacity, and future studies may consider using percentage change thresholds or continuous endpoints. Although this study addressed class imbalance using SMOTE technology, future research could further explore the feasibility of refining efficacy into multi-level or continuous endpoints to more finely characterize the dose-effect relationship of EECP. Third, the predictive factors included in this study mainly focused on baseline clinical characteristics and echocardiographic parameters, without encompassing multi-omics variable information such as serum biomarkers, cardiac magnetic resonance tissue characterization imaging, and genetic variants. With the advancement of the precision medicine concept, incorporating multimodal data into the search space of AutoML in future studies is expected to further enhance the model's prediction accuracy and depth of biological interpretation. Fourth, the follow-up period of this study was concentrated on the evaluation of short-term efficacy after EECP treatment, and systematic assessment of long-term cardiovascular adverse event rates and all-cause mortality has not yet been conducted. Considering the multifactorial nature of long-term prognosis in HFpEF patients, future research should extend the prediction endpoint to the dimension of long-term prognosis, constructing a prediction system covering both short-term efficacy and long-term outcomes.

Looking ahead, this study has the potential and scope for further deepening in the following directions. First, the web-based clinical decision support system developed here could be evaluated in a prospective pragmatic trial to assess its effects on clinical decision-making, patient adherence, and prognosis, thereby facilitating the translation from algorithm development to clinical application. Second, as dynamic monitoring data continuously accumulate during the diagnosis and treatment of heart failure, early physiological response parameters during treatment (such as hemodynamic change indicators after the first EECP treatment) can be introduced into the model as time-series features, constructing a dynamically updated adaptive prediction system to achieve dynamic re-assessment of risk-benefit and timely adjustment of individualized treatment plans during the treatment process. Third, the key predictive factors and their biological pathways revealed by SHAP attribution analysis are expected to provide important clues for the in-depth exploration of the molecular mechanisms of EECP treatment response. Subsequent studies can combine basic experimental research to verify the causal role of relevant pathways in EECP-mediated cardiovascular protective effects. Fourth, the AutoML framework itself is still in a continuous iterative evolution. In the future, the federated learning paradigm can be integrated into multi-center data collaboration scenarios, achieving distributed training and collaborative optimization of the model while protecting data privacy across centers, further enhancing the model's generalizability and clinical applicability.

## 5 Conclusion

In summary, grounded in the clinical need for individualized prediction of EECP treatment efficacy in HFpEF patients, this study constructed a set of AutoML prognostic prediction models driven by an improved metaheuristic optimization algorithm, and validated its predictive performance superior to traditional statistical models and mainstream ensemble learning models within a rigorous methodological framework. Through SHAP interpretability analysis, this study revealed the important predictive roles of echocardiographic and clinical indicators, including age, body mass index, left atrial volume index, left ventricular ejection fraction, and E/e' ratio, in EECP treatment response, providing data-driven clues for understanding the pathophysiological mechanisms of efficacy heterogeneity. Concurrently, the clinical decision support system developed in conjunction with this research has built a bridge for translating precise efficacy assessment from theoretical models to bedside application. The construction and validation of this model provide a interpretable, and clinically actionable intelligent assessment tool for indication screening and individualized decision-making for EECP treatment in HFpEF patients, which is expected to play a positive role in optimizing precision management strategies for this special population.

## Data Availability

The datasets presented in this article are not readily available because data cannot be shared publicly because of patient privacy. A de-identified dataset can be made available from Chongqing University Qianjiang Hospital Institutional Data Access upon reasonable request (contact via lilong@tly.email). Access will be granted to researchers who submit a formal research proposal, provide evidence of appropriate ethics approval for their intended analyses, and sign a data use agreement that prohibits any attempt to re-identify participants or share the data with third parties. Requests to access the datasets should be directed to Xin Wang, lilong@tly.email.
